# Normal Lung Quantification in Usual Interstitial Pneumonia Pattern: The Impact of Threshold-based Volumetric CT Analysis for the Staging of Idiopathic Pulmonary Fibrosis

**DOI:** 10.1371/journal.pone.0152505

**Published:** 2016-03-31

**Authors:** Hirotsugu Ohkubo, Yoshihiro Kanemitsu, Takehiro Uemura, Osamu Takakuwa, Masaya Takemura, Ken Maeno, Yutaka Ito, Tetsuya Oguri, Nobutaka Kazawa, Ryuji Mikami, Akio Niimi

**Affiliations:** 1 Department of Respiratory Medicine, Allergy and Clinical Immunology, Nagoya City University Graduate School of Medical Sciences, Nagoya, Japan; 2 Department of Radiology, Nagoya City University Graduate School of Medical Sciences, Nagoya, Japan; 3 Department of Radiology, Tokyo Medical University, Tokyo, Japan; University of Allabama at Birmingham, UNITED STATES

## Abstract

**Background:**

Although several computer-aided computed tomography (CT) analysis methods have been reported to objectively assess the disease severity and progression of idiopathic pulmonary fibrosis (IPF), it is unclear which method is most practical. A universal severity classification system has not yet been adopted for IPF.

**Objective:**

The purpose of this study was to test the correlation between quantitative-CT indices and lung physiology variables and to determine the ability of such indices to predict disease severity in IPF.

**Methods:**

A total of 27 IPF patients showing radiological UIP pattern on high-resolution (HR) CT were retrospectively enrolled. Staging of IPF was performed according to two classification systems: the Japanese and GAP (gender, age, and physiology) staging systems. CT images were assessed using a commercially available CT imaging analysis workstation, and the whole-lung mean CT value (MCT), the normally attenuated lung volume as defined from −950 HU to −701 Hounsfield unit (NL), the volume of the whole lung (WL), and the percentage of NL to WL (NL%), were calculated.

**Results:**

CT indices (MCT, WL, and NL) closely correlated with lung physiology variables. Among them, NL strongly correlated with forced vital capacity (FVC) (r = 0.92, *P* <0.0001). NL% showed a large area under the receiver operating characteristic curve for detecting patients in the moderate or advanced stages of IPF. Multivariable logistic regression analyses showed that NL% is significantly more useful than the percentages of predicted FVC and predicted diffusing capacity of the lungs for carbon monoxide (Japanese stage II/III/IV [odds ratio, 0.73; 95% confidence intervals (CI), 0.48 to 0.92; *P* < 0.01]; III/IV [odds ratio. 0.80; 95% CI 0.59 to 0.96; *P* < 0.01]; GAP stage II/III [odds ratio, 0.79; 95% CI, 0.56 to 0.97; *P* < 0.05]).

**Conclusion:**

The measurement of NL% by threshold-based volumetric CT analysis may help improve IPF staging.

## Introduction

Idiopathic pulmonary fibrosis (IPF) is the most frequent chronic idiopathic interstitial lung disease in adults. The typical radiological finding of IPF is termed usual interstitial pneumonia (UIP) pattern. UIP pattern is characterized by honeycomb lung and is a remark of disease progression of IPF [1,2]. The definition of UIP pattern has been established in terms of both histopathological features and radiological findings of high-resolution computed tomography (HRCT) [2]. When the known causes of interstitial lung diseases are excluded, pathological evidence is usually not required when radiological UIP pattern is seen on HRCT for the diagnosis of IPF [2]. Radiological UIP pattern is confirmed when all four of the following criteria are fulfilled: 1) subpleural and basal predominance; 2) reticular abnormality; 3) honeycombing with or without traction bronchiectasis; and 4) absence of features inconsistent with UIP pattern, such as micronodules, air trapping, non-honeycomb cysts, extensive ground glass opacities, extensive consolidation, peribronchovascular-predominant distribution, upper lung predominance, or mid-lung predominance [2]

In the last decade, several randomized trials have been conducted to identify efficacious pharmacotherapy for IPF. Pirfenidone [3] and nintedanib [4] are promising drugs that suppress IPF progression. Forced vital capacity (FVC) is the most recommended indicator in staging and monitoring disease progression in IPF. However, a universal staging system that indicates prognosis has not yet been adopted for IPF [2].

Many quantitative methods of computer-aided analysis of chest CT [5–9] have been reported for IPF for quantifying both extension and monitoring progression of the disease. The skewness, kurtosis, and mean lung attenuation calculated from thin-section CT histograms were reported to be correlated with the physiological impairment of this disease [5]. In 2007, development of methods for computer-aided four-class discrimination of the lung region, which includes normal, reticular, honeycombing, and emphysema regions, was reported and this method was called “texture analysis” [6]. Since then, similar texture analysis methods have been reported; studies have indicated their clinical usefulness for the diagnosis of IPF [7], as well as for monitoring its progression [8]. Nevertheless, a simpler method that distinguishes fibrotic lung from normal lung based on the thresholds of CT values was reported to be correlated with pulmonary function tests in diffuse interstitial lung disease [9]. They defined the threshold between functional lung and interstitial lung disease abnormalities as −700 Hounsfield unit (HU) for patients with diffuse interstitial lung disease. Colombi et al. recently reported that the 40th and 80th percentiles of lung density histograms correlated with pulmonary function test results and visual scores obtained from chest CT in IPF [10].

The purpose of this study was to test the correlation between quantitative-CT indices and lung physiology variables and to determine the ability of such indices to predict disease severity in IPF. Normally attenuated lung was defined as between -950 HU and -701 HU, its sensitivity and specificity for detecting disease severity were examined according to two staging systems for IPF: the Japanese [11] and the GAP (gender, age, and physiology) staging systems [12].

## Patients and Methods

### Patients

The institutional review board at the Nagoya City University Graduate School of Medical Sciences approved this retrospective study (approval number H26-1029; September 12, 2014). Informed consent was waived because the data were analysed anonymously. Clinical records of 116 patients with chronic interstitial lung disease who were referred to Nagoya City University Hospital between November 2012 and August 2014, were reviewed retrospectively. Patients with collagen vascular disease, interstitial pneumonia with autoimmune features, occupational lung disease, granulomatous disease, and drug-induced pneumonia were excluded. Patients with concomitant pulmonary fibrosis and emphysema (demonstrating upper-lobe emphysema and lower-lobe pulmonary fibrosis), and upper lobe predominant lung fibrosis were excluded. Furthermore, patients with acute exacerbation of chronic interstitial pneumonia and those who had other comorbidities, such as lung cancer, infectious diseases, or cardiac failure, were excluded. The eligibility criteria included the availability of all results of pulmonary function tests, the 6-min walk test, and arterial blood gas analysis during the 2 weeks before or after HRCT. Consequently, 55 patients with chronic interstitial lung disease were selected. The radiological differential diagnoses made by two radiologists (NK and RM) were compared with the clinical diagnoses by physicians. In the present study, the radiological differential diagnoses were made in 4 categories consisting of IPF, chronic hypersensitivity pneumonia, interstitial pneumonia associated with connective tissue disease, and idiopathic nonspecific interstitial pneumonia, with no knowledge of clinical information. The clinical diagnoses were made by physicians based on clinical courses, symptoms and physiological findings, serum antibodies for antigens that caused hypersensitivity pneumonia, and pathological findings that were available. The numbers of IPF were 32 and 36 by the two radiologists, respectively, and 43 by the physicians. The numbers of chronic hypersensitivity pneumonia were 1 and 2 by the two radiologists, respectively, and 3 by the physicians. The numbers of interstitial pneumonia associated with connective tissue disease were 9 and 3 by the two radiologists, respectively, and 0 by the physicians. The numbers of idiopathic nonspecific interstitial pneumonia were 13 and 14 by the two radiologists, respectively, and 9 by the physicians. The diagnosis of IPF was established according to 2011 international guidelines [2]. Each radiological diagnosis of UIP pattern, possible UIP pattern, and inconsistent with the UIP pattern was made by NK and RM. Healthy persons were recruited as a control group. They had no abnormal findings including nodules, mass, and infiltration on chest CT despite an abnormal shadow on chest X-ray at their medical check- up.

### Automatic derivation of quantitative-CT indices

**Fig 1:**
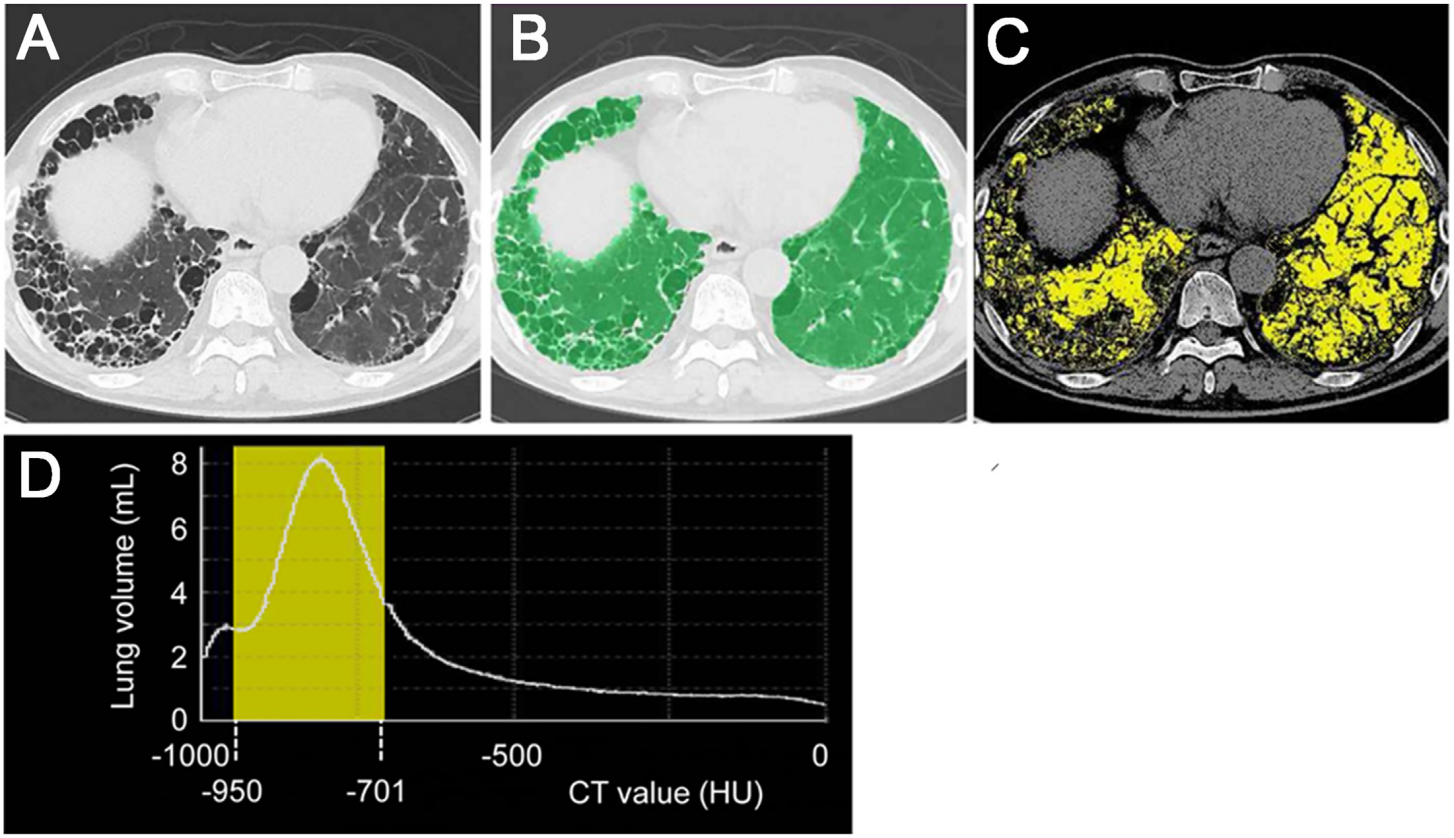
Volumetric analysis of normally attenuated lung. Chest computed tomography (CT) image of a 79-year-old man with IPF showing UIP pattern. This CT slice shows peripheral honeycomb lung predominantly on the right side (A). The CT image showing extracted whole lungs (in green) (B). The volume of the whole lungs (WL) is 2,438 milliliter (mL). The CT image showing normally attenuated lung, as defined between −950 Hounsfield units (HU) and −701 HU (in yellow) (C). The volume of normally attenuated lung (NL) is 1,346 mL. Although some parts of honeycombing are marked as normally attenuated lung (in yellow) by the workstation, most areas of normally attenuated lung are considered to be identical to normal lung tissue. The yellow area under the histogram curve represents the NL (D). The percentage of NL (NL%) is 55.2%. Forced vital capacity and diffusing capacity of the lungs for carbon monoxide of this patient are 1.59 L (52.1%, %predicted) and 6.6 mL/min/mmHg (59.2%, %predicted), respectively. His partial pressure of arterial oxygen is 65.9 Torr, and the lowest percutaneous oxygen saturation is 84% during the 6-min walk test. The patient was classified in Japanese stage IV and GAP stage II.

All patients underwent HRCT using a single CT scan machine (SOMATOM Definition Flash; Siemens Healthcare, Tokyo, Japan). The settings of CT machine were as follows: detector-row configuration acquired as 128 x 0.6 mm by double sampling in the z-direction, 120 kVp, and quality mAs by CARE Dose4D. Mean and standard deviation (SD) of the effective mAs were 176.9 mAs and 44.2 mAs, respectively. The reconstruction algorithm for CT data comprised Sinogram-Affirmed Iterative Reconstruction (SAFIRE), and a sharp kernel of I70. CT images of 2-mm slice thickness for 2-mm intervals in the lung parenchyma [window level: −600 HU; window width: 1500 HU] were used for analysis. All patients underwent CT scan examinations in the supine position at full inspiration without intravenous contrast. Before CT, patients were instructed to maintain full inspiration without coughing during CT. Synapse VINCENT version 3.5 (Fujifilm Medical Systems, Tokyo, Japan) [13,14], a commercially available CT imaging analysis workstation, was used. Digital imaging and communications in medicine (DICOM) data for each patient were transferred to this workstation anonymously. Using this system, whole lung extraction from the chest CT imaging is automatically available by excluding the thoracic wall, mediastinum, large vessels, and airways toward tertiary bronchi (Fig 1A and 1B). This lung extraction was performed by using both threshold values and anatomical knowledge-based algorithms. After this, the observer (HO) checked the automatic lung extraction. The observer is a pulmonary physician with four years of experience checking automated segmentation. In cases that have very large cysts or strong architectural distortion, such as cystic bronchiectasis, whole lungs cannot be extracted correctly by this workstation. In these cases, the extraction of whole lungs can be made by adding manual operation. The mean CT value (MCT) was also available. In addition, threshold-based volumetric CT analysis was available, in which threshold CT values can be flexibly determined by all users. CT values for the whole lung were defined as between -1000 HU and 0 HU. The volume of the extracted whole lungs (WL), the volumes of normally attenuated lung (NL), as defined from −950 HU to −701 HU, and its percentage of the whole lung volume (NL%) were calculated (Fig 1C and 1D).

### Clinical staging of IPF

Clinical staging of IPF was conducted according to two classification systems: the Japanese severity classification system (Table 1) [11] and the GAP staging system (Table 2) [12]. Spirometry was performed using standard methods. Residual volume and total lung capacity (TLC) were measured by the closed-circuit helium method, and diffusing capacity of the lungs for carbon monoxide (DLco) was measured using the single-breath technique. %FVC (percentage of predicted FVC) and %DLco (percentage of predicted DLco) were calculated based on the patients’ height, age, and sex, according to the Japanese standardized methods [15].

**Table 1:** Japanese staging system [11].

Japanese stage	PaO_2_ at rest	SpO_2_ < 90% during 6-min walk test
I	≥ 80Torr	
II	70–79 Torr	stage increased by one (i.e. stage III)
III	60–69 Torr	stage increased by one (i.e. stage IV)
IV	<60Torr	

PaO_2_, partial pressure of arterial oxygen: SpO_2_, percutaneous oxygen saturation

**Table 2:** GAP (gender, age, and physiology) index [12].

	Predictor	Points
**G**	**Gender**	
	Female	0
	Male	1
**A**	**Age years**	
	<61	0
	61–65	1
	>65	2
**P**	**Physiology**	
	FVC % predicted	
	>75	0
	50–75	1
	<50	2
	DLco % predicted	
	>55	0
	36–55	1
	≤35	2
	Cannot perform	3

FVC, forced vital capacity; DLco, diffusing capacity of the lung for carbon monoxide. Patients with total scores of 0–3, 4–5, and 6–8 were classified as GAP stages I, II, and III, respectively.

### Statistical analysis

Statistical analyses were conducted using JMP statistical software (version 11; SAS Institution Japan Ltd, Tokyo, Japan). All data were presented as means and standard deviations (SDs). Continuous and categorical variables were compared between patients with IPF and healthy individuals using Student's t-test and Pearson's chi-square test, respectively. Radiologist agreement was tested by computing Cohen’s kappa coefficient. Pearson product-moment correlation coefficients were used to test correlations of CT imaging indices (MCT, WL, NL, and NL%) and lung physiology variables. Because the numbers of patients in each clinical stage were small, patients with Japanese stage II, Japanese stage III, and Japanese stage IV were combined as a composite stage of Japanese stage II/III/IV. In the same way, the combination of Japanese stage III and Japanese stage IV, GAP stage II and GAP stage III, were collected as Japanese stage III/IV and GAP stage II/III, respectively. Receiver operating characteristic (ROC) curve analysis was performed to examine CT image indices (MCT, WL, NL and NL%) for detecting moderate or more severe stages of IPF. Among these, specificities and sensitivities were calculated for NL%. Multivariable logistic regression analyses were performed and odds ratio and 95% confidence intervals (CI) were calculated to reveal the utility of CT image indices (MCT, WL, NL.and NL%) for the detection of single or composite IPF stages. In the same way, multivariable logistic regression analysis were performed and odds ratio and 95% CI were calculated to make sure the utility of NL% and lung physiology variables for the detection of single or composite stages of IPF. A *P* value less than 0.05 was considered significant.

## Results

### Population characteristics

Each radiological diagnosis of UIP pattern, possible UIP pattern, and inconsistent with the UIP pattern was made by two radiologists. Cohen’s kappa value of the 2 radiologists for these patterns was 0.66. Twenty-seven patients were judged as showing radiological UIP pattern by both two radiologists, and diagnosed as IPF by the physicians. Then, twenty seven patients were enrolled in this study. The clinical and physiological characteristics of the study population are shown in Table 3. The control group was younger than the IPF group (P < 0.001), and predominantly female (P < 0.001). Smoking status and body mass index did not significantly differ between the two groups, whereas FVC (P < 0.001), %FVC (P < 0.001) and FEV_1_ (forced expiratory volume in 1)/FVC (P < 0.05) were lower in the control, than in the IPF group.

**Table 3:** Clinical and physiologic data of the study population.

Parameters	IPF patients	Control
Total (n)	27	13
Gender, male/female (n)	17 / 10	3 / 10
Age (years)	75.5 ± 6.9	59.5 ± 11.8
Never smoker (n)	10	8
Past smoker (n)	17	5
(pack-year)	51 ± 34	23 ± 14.4
Body mass index (kg/m^2^)	22.4 ± 3.7	23.9 ± 3.1
Japanese stage I (n) / II (n) / III (n) / IV (n)	11 / 4 / 9 / 3	NA
GAP stage I (n) / II (n) / III (n)(n)	12 / 6 / 9	NA
Serum KL-6 (U/mL)	1256 ± 1057	NA
Treatment with prednisolone (n)	8	NA
Dose of prednisolone (mg)	11.5 ± 4.9	NA
Treatment with pirfenidone (n)	12	NA
Dose of pirfenidone, 600 mg (n)/1200mg (n)/1800mg (n) 1981800mg (n)	5 / 4 / 3	NA
Home oxygen treatment (n)	7	NA
sPAP assessed with echocardiography >45 Torr (n)	3	NA
FVC (mL)	2303 ± 770	3080 ± 835
%FVC (%)	79.2 ± 18.3	112 ± 20.6
FEV_1_/FVC (%)	84.1 ± 9.9	76.3 ± 8.5
DLco (mL/min/mmHg)	9.1 ± 3.8	NA
%DLco (%)	42.8 ± 16.4	NA
TLC (mL)	3568 ± 1001	NA

Values are presented as mean and standard deviation (SD); GAP stage, mL, milliliter; GAP (gender, age, and physiology) stage: sPAP, systolic pulmonary artery pressure; FVC, forced vital capacity; %FVC, percentage of predicted forced vital capacity, FEV_1_, forced expiratory volume in 1 second; DLco, diffusing capacity of the lungs for carbon monoxide; %DLco, percentage of predicted diffusing capacity of the lungs for carbon monoxide; TLC, total lung capacity. NA, not available.

### Automatic derivation of quantitative-CT indices

Manual operation was not required to extract whole-lungs in the patients enrolled in the present study. The value of MCT for the 27 patients was −755 ± 45.0 HU [mean ± standard deviation (SD)]. The value of WL was 3.4 ± 1.0 L (mean ± SD). The value of NL was 2.2 ± 0.8 L (mean ± SD). The value of NL% was 63.2% ± 9.4% (mean ± SD). This workstation required approximately 2 minutes to compute CT quantitative indices for the data from a single patient.

### Correlations of CT imaging indices with lung physiology variables

**Fig 2:**
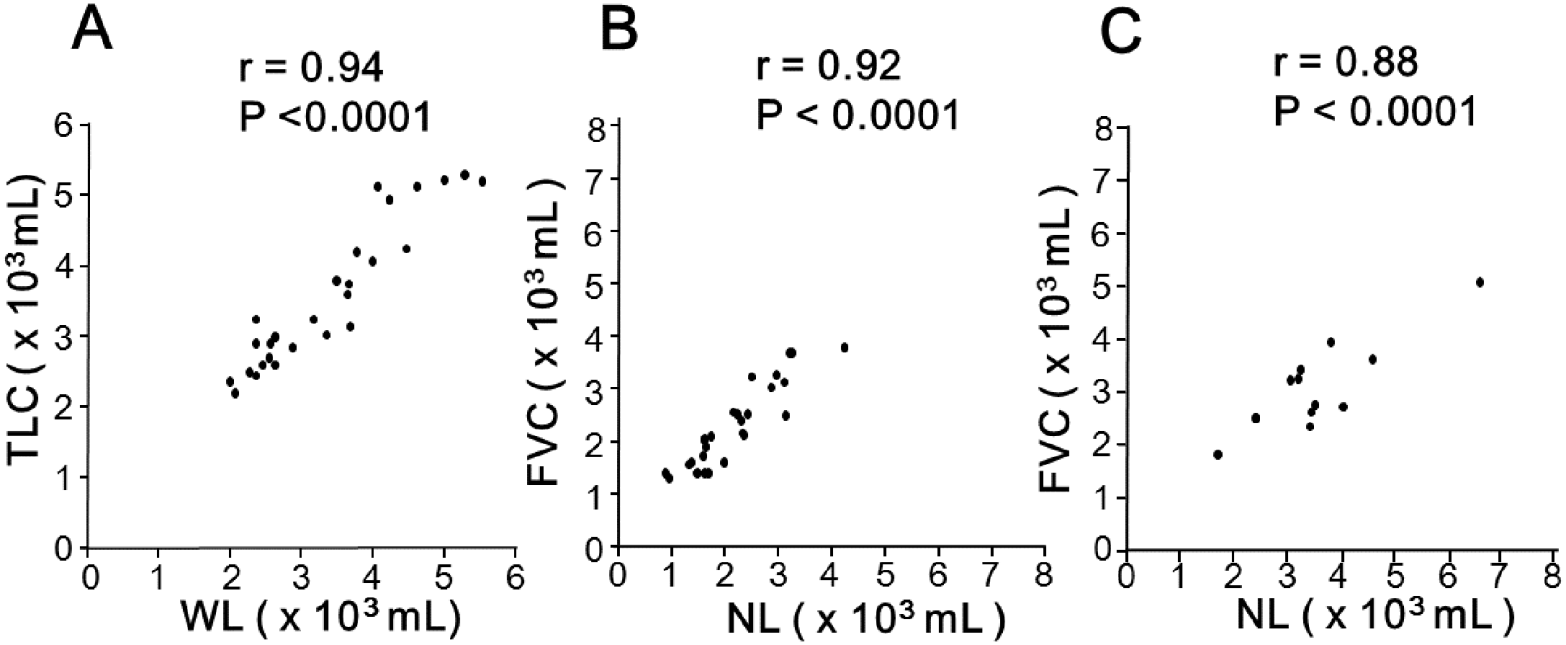
Correlations of CT imaging indices with lung physiology variables. The volume of the whole lungs extracted from CT imaging (WL) is strongly correlated with total lung capacity (TLC) (r = 0.94, *P* < .0001) (A), and the volume of normally attenuated lung (NL) is also well correlated with forced vital capacity (FVC) (r = 0.92, *P* < 0.0001) in patients with IPF (B). The NL was also correlated with FVC in control group (r = 0.88, *P* < 0.0001) (C).

Table 4 shows the correlations between the CT imaging indices and lung physiology variables. Among IPF patients, WL was strongly correlated with TLC (r = 0 .94, *P* < 0.0001) (Fig 2A), and NL was also well correlated with FVC (r = 0.92, *P* < 0.0001) (Fig 2B). NL% was modestly correlated with DLco (r = 0.52, *P* < 0.01) and %DLco (r = 0.55, *P* < 0.01). In the control group, the NL was well correlated with FVC (r = 0.88, *P* < .0001) (Fig 2C).

**Table 4:** Correlations of CT imaging indices with lung physiological variables among IPF patients.

	MCT (HU)		WL (mL)		NL (mL)		NL%	
	*r*	*P* value	*r*	*P* value	*r*	*P* value	*r*	*P* value
FVC (mL)	−0.60	< 0.001	0.96	< 0.0001	0.92	< 0.0001	0.32	0.09
%FVC (%)	−0.66	< 0.001	0.68	< 0.0001	0.61	< 0.0001	0.27	0.16
DLco (mL/min/mmHg)	−0.52	< 0.01	0.72	< 0.0001	0.80	< 0.0001	0.52	< 0.01
%DLco (%)	−0.47	0.01	0.53	< 0.01	0.65	< 0.001	0.55	< 0.01
TLC (mL)	−0.63	< 0.001	0.94	< 0.0001	0.68	< 0.0001	0.31	0.12

HU, Hounsfield unit; mL, millilitre; MCT, mean CT value of whole lungs; WL, volume of the whole lungs from CT imaging; NL, volume of normally attenuated lung from CT imaging, NL%, percentage of the volume of normally attenuated lung in whole lungs extracted from CT imaging

### ROC curve analyses for advanced stage of IPF

**Fig 3:**
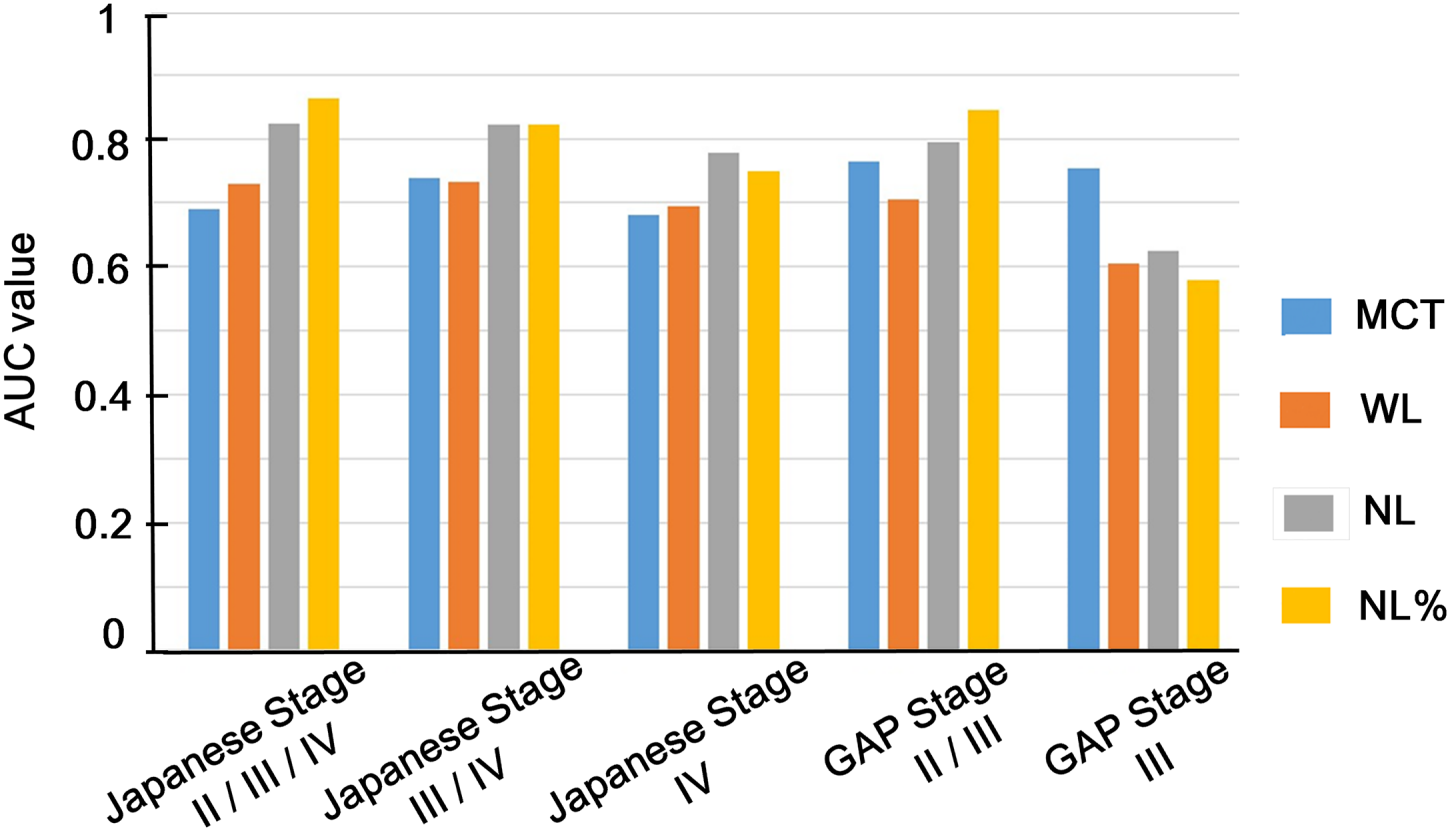
The AUC values of CT imaging indices for clinical staging of IPF. The area under the curve (AUC) values of CT imaging indices, including mean CT value of the whole lungs (MCT), the volume of the whole lungs extracted from CT imaging (WL), the volume of normally attenuated lung (NL), and the percentage of the volume of normally attenuated lung in whole lungs from CT imaging (NL%), are shown.

**Fig 4:**
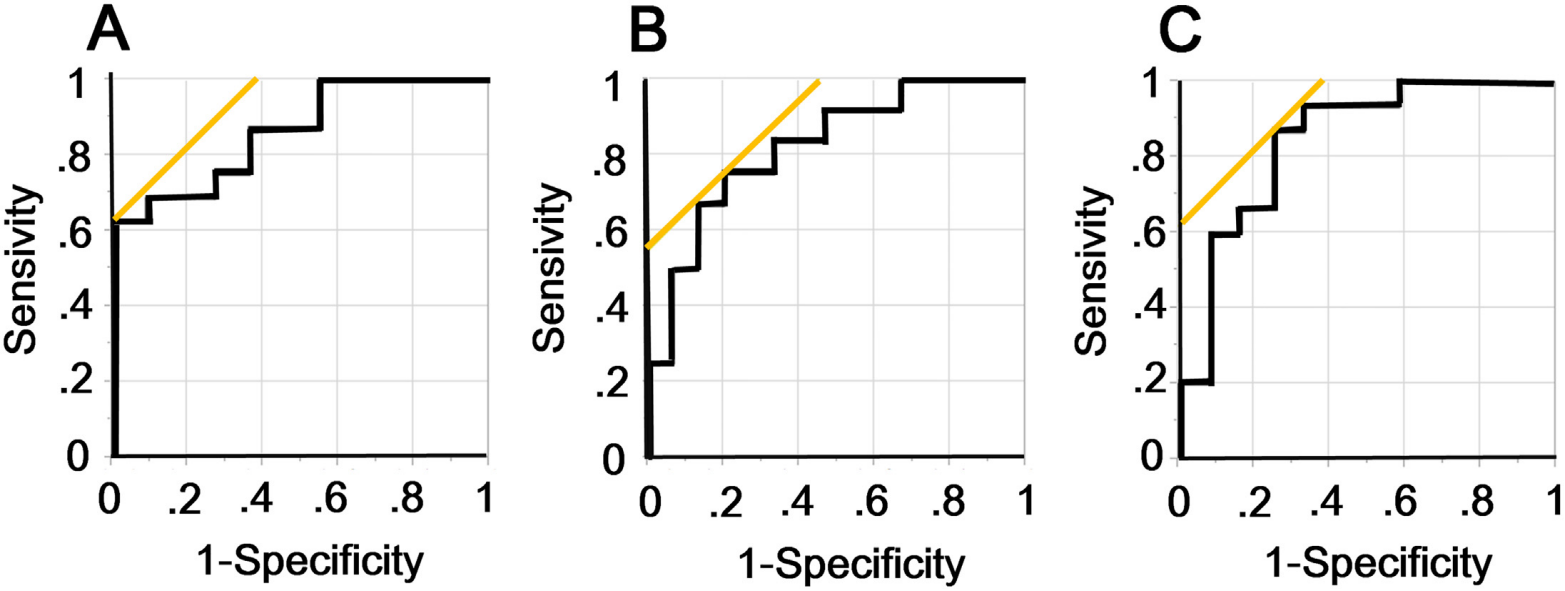
ROC curves of NL%. The receiver operating characteristic (ROC) curves of the percentage of the volume of normally attenuated lung in whole lungs from CT imaging (NL%) for clinical composite unit stages of Japanese stage II/III/IV (A), Japanese stage III/IV (B), and GAP stage II/III (C). The values of AUC are 0.864, 0.822, and 0.844, respectively.

The area under the curve (AUC) values of the ROC curve analysis for the detection of single or composite unit stages in the Japanese and GAP staging systems are shown in Fig 3. The ROC curves of NL% for composite unit stages of Japanese stage II/III/IV (Fig 4A), Japanese stage III/IV (Fig 4B), and GAP stage II/III (Fig 4C) showed that the values of AUC were 0.86, 0.82, and 0.84, respectively. If the cut-off value of NL% was set at 60.4% for detecting Japanese stage II/III/IV, the sensitivity was 62.5%, and the specificity was 100%. If the cut-off value was 63.8% for Japanese stage III/IV, the sensitivity was 75%, and the specificity was 80%. If the cut-off value was 66.0% for GAP stage II/III, the sensitivity was 87%, and the specificity was 75%.

### Multivariable logistic regression analyses for advanced stage of IPF

Multivariable logistic regression analyses were performed for the three parameters of MCT, WL, and NL% (Table 5). NL% was significant for detecting the composite unit stages of Japanese stage II/III/IV (odds ratio, 0.70; 95% CI, 0.47 to 0.89; *P* < 0.001), Japanese stage III/IV (odds ratio, 0.77: 95% CI, 0.57 to 0.93; *P* < 0.01), and GAP stage II/III (odds ratio, 0.75; 95% CI, 0.53–0.91; *P* < 0.001). Furthermore, multivariable logistic regression analyses were performed to compare the NL and NL%. NL% was modestly significant for the detection of Japanese stage II/III/IV (odds ratio, 0.75; 95% CI, 0.51 to 0.94; *P* < 0.01), Japanese stage III/IV (odds ratio, 0.83; 95% CI, 0.64 to 0.99: *P* < 0.05), and GAP II/III (odds ratio, 0.79; 95% CI, 0.59 to 0.97; *P* < 0.05). (Table 6).

**Table 5:** Multivariable logistic regression analyses of CT image indices for single or composite unit stages in the Japanese and GAP staging systems.

	Parameters	Odds ratio	95% CI	*P* value
Japanese stage II/III/IV	MCT	0.99	0.94–1.04	0.71
	WL	0.99	0.99–1.00	0.11
	NL%	0.70	0.47–0.89	< 0.001
Japanese stage III/IV	MCT	1.02	0.98–1.07	0.31
	WL	0.99	0.99–1.00	0.46
	NL%	0.77	0.57–0.93	< 0.01
Japanese stage IV	MCT	0.99	0.96–1.04	0.98
	WL	0.99	0.99–1.00	0.25
	NL%	0.98	0.86–1.13	0.76
GAP stage II/III	MCT	1.02	0.99–1.08	0.23
	WL	0.99	0.99–1.00	0.66
	NL%	0.75	0.53–0.91	< 0.001
GAP stage III	MCT	1.01	0.99–1.04	0.36
	WL	0.99	0.99–1.00	0.82
	NL%	0.99	0.89–1.10	0.87

95% CI: 95% confidence intervals, GAP stage, GAP (gender, age, and physiology) stage; MCT, mean CT value of the whole lung; WL, volume of the whole lungs from CT imaging; NL %, percentage of the volume of normally attenuated lung in whole lungs from CT imaging.

**Table 6:** Multivariable logistic regression analyses of NL and NL % for single or composite unit stages in the Japanese and GAP staging systems.

	Parameters	Odds ratio	95% CI	*P* value
Japanese stage II/III/IV	NL	0.99	0.99–0.99	< 0.05.
	NL%	0.75	0.51–0.94	< 0.01
Japanese stage III/IV	NL	0.99	0.99–1.00	0.06
	NL%	0.83	0.64–0.99	< 0.05
Japanese stage IV	NL	0.99	0.99–1.00	0.15
	NL%	1.03	0.88–1.24	0.70
GAP stage II/III	NL	0.99	0.97–1.00	0.07
	NL%	0.79	0.59–0.97	< 0.05
GAP stage III	NL	1.00	0.99–1.00	0.23
	NL%	0.98	0.87–1.09	0.75

95% CI, 95% confidence intervals; GAP stage, GAP (gender, age, and physiology) stage; NL, volume of normally attenuated lung from CT imaging; NL%, percentage of the volume of normally attenuated lung in whole lungs extracted from CT imaging.

### Multivariable logistic regression analyses of NL% and lung physiology variables

Multivariable logistic regression analyses were performed for the three parameters of NL%, %FVC, and %DLco. The results are shown in Table 7. NL% was significant for detecting the composite unit stages of Japanese stage II/III/IV (odds ratio, 0.73; 95% CI, 0.48 to 0.92; *P* < 0.01), Japanese stage III/IV (odds ratio. 0.80; 95% CI 0.59 to 0.96; *P* < 0.01) and GAP stage II/III (odds ratio, 0.79; 95% CI, 0.56 to 0.97; *P* < 0.05). %DLco was significant for detecting GAP stage III (odds ratio, 0.83; 95% CI, 0.66 to 0.96; *P* < 0.01).

**Table 7:** Multivariable logistic regression analyses of NL % and lung physiology variables for single or composite unit stages in the Japanese and GAP staging systems.

	Parameters	Odds ratio	95% CI	*P* value
Japanese stage II/III/IV	NL%	0.73	0.48–0.92	< 0.01
	%FVC	0.93	0.62–1.02	0.11
	%DLco	1.01	0.91–1.12	0.89
Japanese stage III/IV	NL%	0.80	0.59–0.96	< 0.01
	%FVC	0.94	0.85–1.01	0.07
	%DLco	0.98	0.89–1.09	0.74
Japanese stage IV	NL%	0.94	0.79–1.11	0.43
	%FVC	0.94	0.84–1.02	0.15
	%DLco	1.04	0.94–1.16	0.43
GAP stage II/III	NL%	0.79	0.56–0.97	< 0.05
	%FVC	0.94	0.85–1.02	0.14
	%DLco	0.91	0.79–1.02	0.13
GAP stage III	NL%	1.10	0.96–1.29	0.18
	%FVC	0.97	0.88–1.06	0.52
	%DLco	0.83	0.66–0.96	< 0.01

95% CI: 95% confidence intervals; NL%, GAP (gender, age, and physiology) stage, GAP stage; NL%, percentage of the volume of normally attenuated lung in whole lungs extracted from CT imaging; %FVC, percentage of predicted forced vital capacity, %DLco, percentage of predicted diffusing capacity of the lungs for carbon monoxide.

## Discussion

The present study demonstrated that CT imaging indices from routine CT are well correlated with lung physiology variables among patients with IPF showing radiological UIP pattern. Furthermore, these CT indices had large AUC values for detecting patients in the moderate or advanced stages of IPF. Among these CT image indices, NL% was found to be significantly more useful in detecting some composite unit stages (Japanese stage II/III/IV, Japanese stage III/IV, GAP stage II/III) than %FVC and %DLco. Threshold-based volumetric CT analysis is a simpler method than the previously published texture analysis [6–8]. Taken together, measuring NL% may become useful tool for clinical staging of IPF.

One of the strong points of the present results is that NL was well correlated with FVC. The discovery of this strong correlation is important, because FVC is currently regarded as the most reliable indicator in clinical trials for evaluating pharmacologic treatments [3,4]. It is possible that measuring NL can become an alternative indicator in patients who cannot undergo pulmonary function tests.

Previous studies of CT analysis of lungs defined different thresholds of CT values for the identification of normal lungs. Currently, the CT value of −950 HU is most accepted as the threshold between emphysema and normal lung [16,17]. The area lower than −950HU is called the “low attenuation area (LAA),” with the percentage termed “% LAA” or “LAA%.” Nonetheless, a few reports have been published on the threshold between normal lung and ground glass attenuation. Shin et al. defined the threshold between functional lung and interstitial lung disease as −700 HU for patients with diffuse interstitial lung disease [9]. Kauczor et al. defined ground glass opacity as thresholds between −750 HU and −300 HU for various interstitial lung diseases [18]. In another report, localized ground glass opacity was determined from −740 HU to −174 HU in the investigation of pulmonary nodular lesions [19]. We performed a preliminary analysis of 16 patients with IPF to detect optimal thresholds. Various higher thresholds (-800 HU, -750 HU, -700 HU, -650 HU, and -550 HU) and lower thresholds (-950 HU and -900 HU) were tested. The efficacies of the obtained results for correlations with FVC were similar, but NL calculated by thresholds of -950 HU and -700 HU was regarded as optimal because this became an approximation of FVC (Fig 2B). Therefore, NL was defined in the present study by thresholds of -950 HU and -700 HU based on our results and with reference to the results of previous studies.

In the present study, some of honeycombing lung regions were judged as representing normally attenuated lung. Because honeycombing cysts consist of internal air and surrounding fibrotic walls, the densities of honeycomb cysts are variable due to the partial volume effect of CT with 2-mm slices thickness. This effect is influenced by the size of honeycombing cysts and the axis of the CT slice. The same phenomenon may arise with ground-glass opacities and reticulations. Taking thinner-slice chest CT (0.5 or 1.0 mm) may help reduce partial volume effects. However, most areas of normally attenuated lung were considered to be identical to normal lung tissue in the 2 mm thickness CT slices used in the present study. A good ventilation perfusion ratio would be present in normal lung tissue in IPF patient. From this point of view, our results of the association of NL% with the Japanese staging system is reasonable, because the Japanese staging system is based on partial pressure of arterial oxygen at rest and exertional desaturation.

There are limitations in the present study. First, the eligibility criteria included only IPF patients who showed the radiological UIP pattern. It remains unknown whether similar results can be obtained among patients with IPF who show possible UIP pattern and inconsistent UIP pattern on CT. Second, the results were obtained by retrospective analysis of a small number of patients from a single center. Composite disease severity stages were formed due to a small number of patients was present in each stage. Merging stages of the Japanese and GAP classification systems with significantly different mortality rates is a limitation of this study. Third, the threshold values that determine “normally attenuated lung” may differ with each CT machine and the conditions, including slice thickness, pitch, and reconstruction algorithm. The combination of a large slice thickness and a large reconstruction interval (2-mm slice thickness and 2-mm reconstruction interval) with a sharp kernel (I70) is a limitation of the automatic quantitative analysis of lung in the present study. Fourth, we did not use spirometric gating to achieve constant full-breath-hold inspiration in all patients, and this might have affected CT density. Therefore, the data of this study should be interpreted as preliminary.

## Conclusion

NL% was found to be significantly more useful in detecting some composite unit stages (Japanese stage II/III/IV, Japanese stage III/IV) than %FVC and %DLco. Further research with large numbers of patients is required to examine whether measuring NL% improve the quality of the clinical staging of IPF to indicate disease severity, disease monitoring, and prognosis.
