# Correction: Normal Lung Quantification in Usual Interstitial Pneumonia Pattern: The Impact of Threshold-based Volumetric CT Analysis for the Staging of Idiopathic Pulmonary Fibrosis

**DOI:** 10.1371/journal.pone.0160231

**Published:** 2016-08-04

**Authors:** Hirotsugu Ohkubo, Yoshihiro Kanemitsu, Takehiro Uemura, Osamu Takakuwa, Masaya Takemura, Ken Maeno, Yutaka Ito, Tetsuya Oguri, Nobukata Kazawa, Ryuji Mikami, Akio Niimi

The ninth author’s name is spelled incorrectly. The correct name is: Nobukata Kazawa.

In Table 3 the description of the dose of pirfenidone is incorrect. The correct dose of pirfenidone is: 600 mg (n)/1200mg(n)/1800mg(n). Please see the corrected Table 3 here.

**Table 3:** Clinical and physiologic data of the study population.

Parameters	IPF patients	Control
Total (n)	27	13
Gender, male/female (n)	17 / 10	3 / 10
Age (years)	75.5 ± 6.9	59.5 ± 11.8
Never smoker (n)	10	8
Past smoker (n)	17	5
(pack-year)	51 ± 34	23 ± 14.4
Body mass index (kg/m^2^)	22.4 ± 3.7	23.9 ± 3.1
Japanese stage I (n) / II (n) / III (n) / IV (n)	11 / 4 / 9 / 3	NA
GAP stage I (n) / II (n) / III (n)	12 / 6 / 9	NA
Serum KL-6 (U/mL)	1256 ± 1057	NA
Treatment with prednisolone (n)	8	NA
Dose of prednisolone (mg)	11.5 ± 4.9*	NA
Treatment with pirfenidone (n)	12	NA
Dose of pirfenidone, 600 mg (n)/1200mg (n)/1800mg (n)	5 / 4 / 3	NA
Home oxygen treatment (n)	7	NA
sPAP assessed with echocardiography >45 Torr (n)	3	NA
FVC (mL)	2303 ± 770	3080 ± 835
%FVC (%)	79.2 ± 18.3	112 ± 20.6
FEV_1_/FVC (%)	84.1 ± 9.9	76.3 ± 8.5
DLco (mL/min/mmHg)	9.1 ± 3.8	NA
%DLco (%)	42.8 ± 16.4	NA
TLC (mL)	3568 ± 1001	NA
